# Effect of combined strength and endurance training in adults with asthma: a randomized controlled trial

**DOI:** 10.36416/1806-3756/e20250009

**Published:** 2025-11-14

**Authors:** Giuseppe Lo Bello, Federico Mattia Oliva, Alberto Malovini, Nicolino Ambrosino, Matteo Tarasconi, Andrea Zanini, Elisabetta Zampogna

**Affiliations:** 1. Division of Pulmonary Rehabilitation, Istituti Clinici Scientifici Maugeri IRCCS, Tradate, Italy.; 2. Department of Anesthesia and Intensive Care, IRCCS San Raffaele Scientific Institute, Milan, Italy.; 3. Laboratory of Medical Informatics and Artificial Intelligence, Istituti Clinici Scientifici Maugeri IRCCS, Pavia, Italy.; 4. Istituti Clinici Scientifici Maugeri IRCCS, Respiratory Rehabilitation of the Institute of Montescano, Montescano, Pavia, Italy.; 5. Pulmonary Rehabilitation, Cliniche di Riabilitazione Ente Ospedaliero Cantonale (CREOC), Novaggio, Switzerland.

**Keywords:** Rehabsilitation, Asthma, Exercise therapy, Resistance training, Exercise tolerance, Quality of life

## Abstract

**Objective:**

Pulmonary rehabilitation programs, including exercise training, have an established role in the treatment of chronic respiratory diseases but are not routinely used in asthma. Most studies of individuals with asthma have focused on endurance training, and there is therefore limited data available on strength training. The aim of this study was to evaluate the effects that adding strength training to a program of endurance training and education has on the quality of life of such individuals.

**Methods:**

In this single-center, parallel-group randomized controlled trial, adults with moderate-to-severe asthma admitted for in-hospital pulmonary rehabilitation between June of 2021 and October of 2022 were randomized to either a study group (SG) or a control group (CG). The SG received strength training alongside endurance training and education, whereas the CG received the same endurance training and education, along with sham mobility exercise training instead of strength training. The primary outcome was the change in the Asthma Quality of Life Questionnaire (AQLQ) score from hospital admission to discharge.

**Results:**

A total of 61 participants were randomized, with 31 being assigned to the SG and 30 being assigned to the CG. At discharge, the AQLQ score showed significant improvement in both groups (p < 0.001 for the SG and p = 0.02 for the CG), albeit without a significant difference between the groups (p > 0.99). In contrast, peripheral muscle strength improved significantly from admission to discharge only in the SG, with a significant difference between the groups in terms of quadriceps strength (p = 0.03).

**Conclusions:**

Adding strength training to endurance training and education does not seem to result in further improvement in the quality of life of individuals with moderate-to-severe asthma.

## INTRODUCTION

Asthma is one of the most common chronic respiratory diseases, affecting an estimated 262 million people globally in 2019.^1^ Although drug therapy is effective in most cases, the disease can remain less than optimally controlled in some cases, partly because of incorrect usage of or reduced adherence to pharmacological treatment.^2^ These individuals might not be able to perform activities of daily living and can suffer from poor health-related quality of life. This highlights the need for additional, nonpharmacological interventions,^3^ and exercise training could be a highly effective strategy. In particular, studies showed that exercise training is associated with a reduction in symptoms, improved asthma control, better lung function, and enhanced quality of life.^4^ ^,^ ^5^ Nevertheless, although pulmonary rehabilitation programs that include exercise training have an established role in the treatment of chronic respiratory diseases, they are not routinely employed in individuals with asthma and there are no specific recommendations on the intensity, frequency, or duration of the exercise.^6^ ^-^ ^8^ However, the latest update to the GINA guidelines on asthma management and prevention recommends that individuals with asthma and reduced functional capacity be referred to a pulmonary rehabilitation program.^9^ Although more research is needed to establish the optimal exercise regimen and strong recommendations are currently unavailable, endurance training (ET) is the most widely studied and recommended exercise modality for individuals with asthma.^8^ ^-^ ^11^ Strength training (ST), which involves repetitive lifting of increasing loads to strengthen muscle groups, is another exercise modality recognized for its importance in promoting healthy aging.^12^ ^-^ ^14^ In addition, ST is known to improve not only muscle strength in the limbs, hand grip, and depression but also quality of life in older people,^14^ as well as being indicated in individuals with chronic respiratory diseases.^6^ However, there are few data on the effect of ST on quality of life in individuals with asthma. Therefore, the aim of this trial was to evaluate the short- and long-term effects that adding ST to a program of ET and education has on quality of life in individuals with moderate-to-severe asthma.

## METHODS

This was a single-center, parallel-group randomized controlled trial, approved by the Research Ethics Committee of the *Istituti Clinici Scientifici Maugeri* (Reference no. 2525 CE 08-June-2021), in the city of Tradate, Italy. The study was conducted in accordance with the principles outlined in the Declaration of Helsinki. Participants gave written informed consent, and data were treated confidentially. The study was registered with ClinicalTrials.gov (identifier: NCT04935125; http://www.clinicaltrials.gov/). The reporting adhered to the Consolidated Standards of Reporting Trials guidelines.^15^ The interventions were described following the Consensus on Exercise Reporting Template (CERT).^16^

We evaluated all individuals with asthma, diagnosed in accordance with the GINA guidelines,^9^ who were admitted to the *Istituti Clinici Scientifici Maugeri* Rehabilitation Hospital of Tradate for in-hospital pulmonary rehabilitation between June of 2021 and October of 2022. The inclusion criteria were as follows: being between 18 and 80 years of age; having severe asthma, as defined by GINA steps 4 and 5 under inhalation therapy^9^; presenting with symptoms, as evidenced by an Asthma Control Test score between 20 and 24^17^; and being able to perform and complete the study procedures and the pulmonary rehabilitation program. Subjects were excluded if they met any of the following criteria: having COPD; being a current or former smoker with a smoking history of more than 10 pack-years; having a BMI ≥ 30 kg/m^2^; having a change in medication within the last 30 days before randomization; presenting with cognitive impairment, as evidenced by a Mini-Mental State Examination score < 22^18^; and having a history of cancer, neurological disorder, cardiovascular disease, musculoskeletal impairment, or any condition that would preclude exercise testing and pulmonary rehabilitation.

After baseline evaluations, the eligible participants were enrolled. The randomization list, with a 1:1 ratio, was computer-generated by an independent statistician using dedicated software (https://www.randomizer.org/). Allocation to the study group (SG) or control group (CG) was determined by a researcher not involved in the study, who drew sealed, opaque envelopes, each containing a group code. Participants in the SG underwent ET and education with the addition of ST, whereas those in the CG underwent the same ET and education program along with a sham intervention, which consisted of unloaded exercises for the upper and lower limbs.

### Measurements

The following data and assessments were recorded or performed at admission, designated time zero (T0): demographics; anthropometrics; asthma severity according to the GINA guidelines^9^; comorbidities, assessed with the Cumulative Illness Rating Scale Severity and Comorbidity Index^19^; the Asthma Control Test score^17^; steroid use and number of exacerbations in the previous 12 months; dynamic lung volumes according to standards^20^ using the predicted values established by Quanjer et al.^21^; arterial blood gases; and airway inflammation, identified by measuring the fractional exhaled nitric oxide (FeNO).^22^ At discharge (T1), we also applied the Global Perceived Effect scale.^23^

At T0, at T1, and at 12 months after discharge (T2), the following were evaluated^24^ ^-^ ^29^:

- Health-related quality of life, by application of the Asthma Quality of Life Questionnaire (AQLQ)
- Disease control, as characterized by the score on the six-item Asthma Control Questionnaire (ACQ-6)
- Functional capacity, as determined by the distance covered on the six-minute walk test; that is, the six-minute walk distance (6MWD)
- Isometric maximal voluntary contraction (MVC) of the quadriceps and biceps, as assessed with a hand-held dynamometer

At T2, the number of exacerbations during the previous 12 months was recorded. Exacerbations were defined as a progressive increase in symptoms and decrease in lung function, requiring a change in medications.^30^ The assessors who conducted the evaluations were blinded to the group allocation. Details of the measurements, including their minimal clinically important difference (MCID) values for outcome measures, are shown in the supplementary material.

### Pulmonary rehabilitation

All interventions were supervised by a team consisting of chest physicians, nurses, physical therapists, dieticians, and psychologists. 

The ET program consisted of 14 daily 30-min sessions (six days per week) of supervised incremental cycling on a cycle ergometer (Ergoselect 4 or Ergoselect 5; Ergoline GmbH, Bitz, Germany). The initial workload was set at 50-70% of the maximal load, calculated on the basis of the baseline 6MWD, as described by Hill et al.^31^ The progression was individualized based on perceived effort: if participants rated their dyspnea or leg fatigue as < 4 on the modified Borg scale,^32^ the workload was increased by 5 watts; if their Borg scale score was 4 or 5, the workload remained unchanged; and if their Borg scale score was > 5, the workload was reduced. Peripheral oxygen saturation, heart rate, arterial blood pressure, perceived dyspnea, and perceived fatigue were monitored during sessions. The total weekly ET volume was 180 min.

The ST program targeted peripheral limb muscle and was performed six times per week in 30-min supervised sessions. Each participant completed the same exercise protocol, performing three sets per exercise, beginning with 8 repetitions per set for the first 4-5 days, then increasing to at least 12 repetitions per set on the following days. The initial load was set to induce moderate fatigue or dyspnea (Borg scale score of 3 or 4). The progression was based on perceived effort: if participants rated their fatigue or dyspnea as < 4 on the Borg scale, the resistance was increased by 0.5-1.0 kg; if the Borg scale score was 4 or 5, the load remained unchanged; and if the Borg scale score was > 5, the resistance was reduced. The training session was conducted one-on-one under the supervision of a physical therapist. The weekly training volume was adjusted based on tolerance and performance, ensuring a progressive overload approach.

The sham training consisted of unloaded mobilization exercises for the upper and lower limbs, performed in small groups under the supervision of a physical therapist. Sessions lasted 30 min, six times per week, and each exercise was performed for three sets of 8-12 repetitions, without any progression in load or intensity.

Additional details on the exercise training modalities can be found in the supplementary material.

Education, provided by chest physicians, nurses, and physical therapists, consisted of at least three individual 20-min sessions on asthma characteristics, drug/inhalation therapy, physical activity, and lifestyle. In addition, a minimum of three 45-min group sessions on diet and nutrition, anxiety, depression and stress control,^33^ were provided by a dietitian and a psychologist.

Full treatment adherence was defined as 80% participation, as assessed by counting the number of sessions completed by each subject.

Before discharge, each subject received written instructions on how to behave at home and how to maintain their exercise training. Specifically, all patients were instructed to perform ET, with ST for those in the SG and sham mobility exercises for those in the CG (more details in the supplementary material).

### Statistical analysis

The primary outcome measure of this study was the change in the AQLQ score from T0 to T1. Secondary outcome measures were the changes in the ACQ-6 score, in the 6MWD, in the quadriceps MVC, and in the biceps MVC-between T0 and T1 and between T0 and T2-in both groups.

The sample size calculation was based on the primary outcome of the study. Data from 29 subjects per group were required to test the null hypothesis of no difference in the AQLQ score from T0 and T1 between the SG-expected mean change, 1.03 ± 0.62 points^34^-and the CG-expected mean change, 0.52 ± 0.41 points^35^-with a significance level of α = 0.05 and a statistical power (1 − β) of 0.95 (two-tailed t-test for independent samples). The total sample size of 58 subjects was rounded up to 60. Sample size calculations were performed with the G*Power software tool, version 3.1.9.2 (Heinrich-Heine-Universität, Düsseldorf, Germany).

All participants were included in the intention-to-treat analysis. Participants who completed the program with available measures at all-time points were included in the per-protocol analysis. Quantitative variables are expressed as mean and standard deviation, or as median and interquartile range when their distribution deviated significantly from the normality assumptions (p < 0.05 on the Shapiro-Wilk test) or in the case of discrete numeric variables. Categorical variables are expressed as absolute and relative frequency. Linear quantile mixed models were applied to estimate the change over time in outcomes between time points and to compare estimates between the SG and CG. The Bonferroni correction was applied to adjust for multiple comparisons. The significance level was set at α = 0.05. Statistical analyses were performed with R software, version 4.2.2 (www.r-project.org). A detailed description of the statistical methods can be found in the supplementary material.

## RESULTS

**Figure 1:**
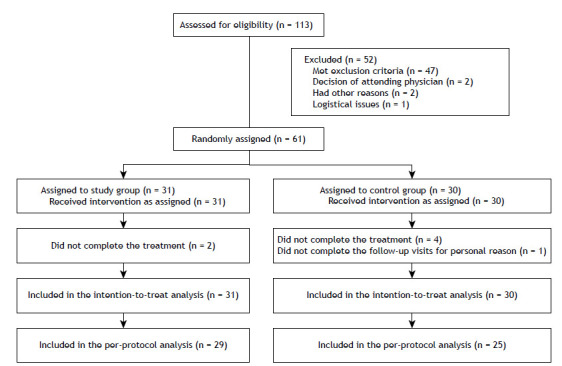
Consolidated Standards of Reporting Trials flow diagram.

We evaluated 61 participants, 31 in the SG and 30 in the CG. Although all participants were included in the intention-to-treat analysis, 7 were excluded from the per-protocol analysis (Figure 1). As shown in Table 1, there were no significant differences between the two groups in terms of the demographic, anthropometric, physiological, or clinical characteristics, or in terms of the medications used. The only statistically significant difference was in the FeNO, which was higher in the SG. In addition, none of the enrolled subjects had any clinically significant respiratory conditions other than asthma.

**Table 1:** Baseline characteristics of the participants.

Variable	Study group	Control group	p
	(n = 31)	(n = 30)	
Male	9 (29)	4 (13)	0.21
Age, years	63 (58-71)	62 (54-72)	0.72
BMI, kg/m^2^	27.1 (23.6-29.0)	26.3 (21.0-29.0)	0.54
GINA step 5	21 (68)	14 (48)	0.20
ACT score	20 (17-21)	20 (16-21)	0.97
Current or former smoker	10 (32)	5 (17)	0.24
Smoking history, pack-years	0 (0-1)	0 (0-0)	0.28
CIRS Severity Index score	1.5 (1.3-1.8)	1.7 (1.4-1.9)	0.19
CIRS Comorbidity Index score	3 (2-5)	4 (2-6)	0.43
Previous exacerbation, n	0 (0-1)	0 (0-1)	0.44
At least one exacerbation in the previous year	12 (39)	14 (47)	0.53
Steroid use in previous 12 months, mg	0.00 (0.00-0.56)	0.00 (0.00-1.08)	0.33
FEV_1_, % pred	85.13 ± 21.53	86.45 ± 24.17	0.82
FVC, % pred	95.39 ± 15.52	92.45 ± 15.35	0.46
PaO_2_, mmHg	80.97 ± 9.79	81.19 ± 12.34	0.94
PaCO_2,_ mmHg	37.3 (35.3-39.6)	37.2 (35.7-41.1)	0.40
pH	7.41 (7.41-7.43)	7.42 (7.40-7.43)	0.99
FeNO^a^, ppb	40.5 (21.8-88.5)	23 (15.3-37.5)	0.01
AQLQ score	5.75 (5.08-6.04)	5.20 (4.38-5.90)	0.16
ACQ-6 score	1.33 (0.50-1.83)	1.42 (0.74-1.66)	0.66
6MWD, m	483.9 ± 71.9	461.9 ± 99.0	0.33
6MWD, % pred	85.1 ± 11.2	79.5 ± 17.3	0.15
Isometric MVC, kg			
Biceps	13.7 (11.8-16.4)	12.3 (9.3-15.1)	0.21
Quadriceps	22.3 ± 7.1	22.4 ± 8.4	0.95

Data are reported as n (%), mean ± SD, or median (IQR). ACT: Asthma Control Test; CIRS: Cumulative Illness Rating Scale; FeNO: fractional exhaled nitric oxide; AQLQ: Asthma Quality of Life Questionnaire; ACQ-6: six-item Asthma Control Questionnaire; 6MWD: six-minute walk distance; and MVC: maximal voluntary contraction. ^a^ Data available for only 52 subjects (26 in the study group and 26 in the control group).

At T0, 4 of the participants in the SG had very high FeNO values (> 100 ppb), which were not observed in any of the participants in the CG. Values exceeding the threshold for eosinophilic inflammation, defined as 25 ppb,^22^ were observed in 17 (55%) of the participants in the SG and in 12 (40%) of those in the CG, with no significant difference between the groups (p = 0.16). Because of technical and organizational constraints, in comparison with the registered protocol, the results of the assessment of the FeNO at T1 and T2 were registered in very few participants, as were those of the cardiopulmonary exercise testing at T0, T1, and T2. Therefore, those data are not reported or commented upon.

The mean number of ET sessions completed was 12 (IQR: 11-15) in the SG and 12 (IQR: 12-14) in the CG (p = 0.92). Participants in the SG also completed 13 ST sessions (IQR: 11-14), whereas those in the CG completed 13 sham training sessions (IQR: 7-14), and the difference was not significant (p = 0.47). Full treatment adherence, defined as completing at least 80% of the 14 sessions, was achieved by 24 (77%) of the participants in the SG and by 20 (67%) of those in the CG, without any statistically significant difference between groups (p = 0.35). No adverse events occurred in either group over the course of the study.

### Primary outcome measure

As shown in Table 2, the AQLQ score improved significantly from T0 to T1 in both groups, without any statistically significant difference between the groups (p > 0.99). The number of participants reaching the MCID on the AQLQ was 18 (62%) in the SG and 12 (46%) in the CG (p = 0.28). 

**Table 2:** Results from linear quantile mixed models according to the intention-to-treat analysis.

Outcome measure(s)	Study group		Control group		Interaction p-value^c^
	(n = 31)		(n = 30)		
	Estimate (95% CI)^a^	p^b^	Estimate (95% CI)^a^	p^b^	
Primary					
Δ T0-T1					
AQLQ score	0.63 (0.27 to 0.98)	< 0.001	0.55 (0.07 to 1.02)	0.02	> 0.99
Secondary					
Δ T0-T1					
ACQ-6 score	−0.69 (−1.18 to −0.21)	0.002	−0.74 (−1.08 to −0.40)	< 0.001	> 0.99
6MWD, m	31.04 (9.79 to 52.28)	0.001	41.21 (16.64 to 65.78)	< 0.001	0.81
6MWD, % pred	6.55 (2.87 to 10.23)	< 0.001	5.93 (2.02 to 9.84)	< 0.001	> 0.99
Isometric MVC, kg					
Quadriceps	6.27 (3.65 to 8.90)	< 0.001	2.35 (−0.77 to 5.47)	0.24	0.03
Biceps	2.39 (0.47 to 4.30)	0.008	1.98 (−0.16 to 4.12)	0.08	> 0.99
Δ T0-T2					
AQLQ score	0.01 (−0.37 to 0.39)	> 0.99	0.36 (−0.20 to 0.91)	0.43	0.36
ACQ-6 score	−0.31 (−0.71 to 0.10)	0.23	−0.53 (−1.00 to −0.05)	0.02	0.72
6MWD, m	17.04 (−14.97 to 49.04)	0.72	27.41 (−10.08 to 64.90)	0.27	> 0.99
6MWD, % pred	2.53 (−2.58 to 7.65)	0.85	2.87 (−3.11 to 8.86)	0.91	> 0.99
Isometric MVC, kg					
Quadriceps	−0.16 (−3.22 to 2.91)	> 0.99	−2.24 (−5.84 to 1.37)	0.48	0.50
Biceps	−0.01 (−2.01 to 1.98)	> 0.99	−0.75 (−2.83 to 1.32)	> 0.99	> 0.99
Exacerbations, n	−0.17 (−0.65 to 0.32)	0.88	−0.63 (−1.32 to 0.06)	0.08	0.18

T0: time zero (admission); T1: time one (discharge); T2: time two (12 months after discharge); AQLQ: Asthma Quality of Life Questionnaire; ACQ-6: six-item Asthma Control Questionnaire; 6MWD: six-minute walk distance; and MVC: maximal voluntary contraction. ^a^Change between time points estimated by linear quantile mixed models and corresponding Bonferroni-corrected 95% CI, adjusted by four tests (AQLQ; ACQ-6; 6MWD, m; 6MWD, % pred; biceps MVC; and quadriceps MVC) or by two tests (exacerbations).^b^Bonferroni-corrected p-value, adjusted by four tests (AQLQ; ACQ-6; 6MWD, m; 6MWD, % pred; biceps MVC; and quadriceps MVC) or by two tests (exacerbations), corresponding to the estimate deriving from linear quantile mixed models.^c^Bonferroni-corrected p-value, adjusted by two tests (AQLQ; ACQ-6; 6MWD, m; 6MWD, % pred; biceps MVC; and quadriceps MVC) or unadjusted (exacerbations) for the interaction between time and group.

### Secondary outcome measures

**Figure 2:**
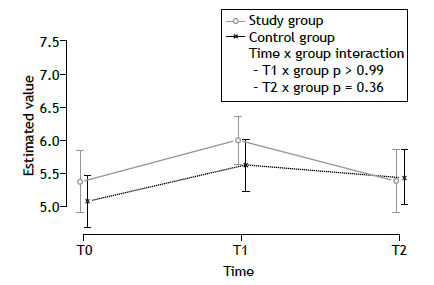
Estimated Asthma Quality of Life Questionnaire scores, by group and time point, in the intention-to-treat analysis. Points show the conditional medians from linear quantile mixed model regression, with the bars representing 95% confidence intervals (no Bonferroni correction). T0: time zero (admission); T1: time one (discharge); and T2: time two (12 months after discharge).

The improvement gained in AQLQ scores by T1 was lost in both groups by T2 (Figure 2). From T0 to T1, the quadriceps and biceps MVC values improved significantly only in the SG participants. In particular, the change estimated in terms of quadriceps MVC was significantly higher in the SG than in the CG (interaction p = 0.03). From T0 to T1, all other outcomes improved significantly in both groups, although without statistically significant differences between the groups. Such benefits were lost by T2 (Table 2 and Supplementary Figures S1-S5). At T1, the median score on the Global Perceived Effect scale was 2 (IQR: 1-2) in the SG and in the CG, with no significant difference between the two groups (p = 0.65). During the year following randomization, there was no significant change in the exacerbation rate in either group. The results adjusted for baseline values (Figures S1-S7 and Table S1) and obtained from the per-protocol analysis (Tables S2 and S3) were similar to those obtained from the unadjusted intention-to-treat analysis. 

**Figure 3:**
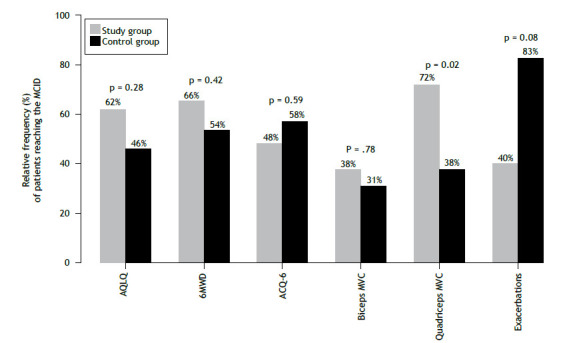
Relative frequency of patients reaching the minimal clinically important difference (MCID) for each outcome, in the study group (n = 31) and in the control group (n = 30). AQLQ: Asthma Quality of Life Questionnaire; 6MWD: six-minute walk distance; ACQ-6: six-item Asthma Control Questionnaire; and MVC: (isometric) maximal voluntary contraction. Frequency of patients reaching the MCID at discharge, for each outcome, except for exacerbations, which were evaluated at 12 months after discharge. Exacerbations were analyzed in a subset of 22 subjects who had at least one exacerbation in the 12 months before randomization and available data on exacerbations during the 12 months after randomization. Values of p are from chi-square tests.

Figure 3 shows the proportion of participants reaching the MCID for each outcome. A significant difference was found only for quadriceps MVC: the proportion of participants reaching the MCID was significantly higher in the SG (p = 0.02).

## DISCUSSION

To our knowledge, this is the first randomized controlled trial to evaluate the effects of adding ST to a program of ET and education in individuals with moderate-to-severe asthma. We found an improvement in the primary and secondary outcome measures in the SG and the GC at T1, with no significant differences between groups, except for the quadriceps and biceps MVC, which improved only in the SG. However, the observed benefits of training were lost by one year after randomization in both groups.

We have assessed individuals with moderate-to-severe asthma according to the GINA guidelines.^9^ It has been shown that individuals with asthma at any GINA step can benefit from pulmonary rehabilitation programs including ET.^36^ Our study is unique in that it adds evidence to support pulmonary rehabilitation programs that also include ST, at least for individuals with moderate-to-severe asthma. Another original result of our study is the improvement in peripheral muscle strength, as evidenced by greater biceps and quadriceps MVC, in the participants receiving ET plus ST but not in those undergoing ET without ST. This result shows the specificity of the training programs we used. To determine the workload for the ST, we adopted a symptom-driven approach rather than the more commonly used method based on a percentage of the one-repetition maximum.^7^ As well as being simpler and more pragmatic, this approach allows the training intensity to be more individualized, enhancing adherence and optimizing functional adaptations. That may have contributed to the observed improvements in muscle strength, because individual adjustments ensured an appropriate yet challenging workload for each participant. All other outcomes improved in both groups. The lack of statistically significant between-group differences in terms of the post-program changes in outcome measures is not surprising given that both groups performed ET, which is associated with improvements in those outcomes.^36^ In addition, the lack of significant differences in the scores on the AQLQ may be partly due to the broad nature of its questions, which assess muscle strength only indirectly, through a few items on physical limitations in daily life. However, this is a common feature of quality-of-life questionnaires, where such aspects are generally assessed only marginally.

In the present study, the benefits observed at T1 were lost by T2, despite the fact that each participant received a written maintenance program at T1. That finding confirms what has been reported in previous studies.^37^ Recent technological advances may facilitate the organization of such programs.^38^

The lack of changes in exacerbation rates in the year following the program in both groups is not surprising. Given the very low rate in the year before the study, further improvement would be highly improbable in a population also enrolled in an educational program.

We are confident that the duration of the program applied in the present study (at least 12 training sessions) was sufficient to reach the plateau of exercise tolerance, as previously shown in individuals with COPD admitted for in-hospital pulmonary rehabilitation.^39^ In fact, a study comparing the functional benefits and relative costs of a short-term intensive inpatient pulmonary rehabilitation program with those of a longer outpatient program for individuals with chronic airway obstruction, concluded that the shorter inpatient program provides improvements in exercise tolerance similar to those of the longer outpatient program, but at a lower cost.^39^

Our study has some limitations. First, there was a statistically significant difference between the SG and CG in terms of the FeNO at baseline. That can be explained by the presence of a few SG participants with very high FeNO values, despite the fact that the proportion of participants in whom eosinophilic inflammation exceeded the 25 ppb threshold did not differ significantly between the two groups. In addition, a recent meta-analysis concluded that FeNO-guided asthma treatment probably results in fewer exacerbations but may not have clinically relevant effects on other asthma outcomes.^40^ Therefore, we are confident that this difference has not biased our results. Furthermore, the use of anklets and wristbands in place of gym machines (unavailable at our facility), particularly for the lower extremity exercises, may have limited the effectiveness of the ST. However, the combination of simple, low-cost equipment and the detailed description of the treatment following the CERT guidelines^16^ makes our program easily replicable and adaptable, even in home or resource-limited settings. Another limitation of our study is the use of the exacerbation rate as the only long-term outcome measure. However, collecting additional relevant outcomes, such as health care resource utilization and lost workdays, would have required a health care data collection system that is not readily available in our country. Finally, the single-center design may have limited the generalizability of the results. However, the use of nonparametric statistical methods and repeated analyses, employing both intention-to-treat and per-protocol approaches, with and without adjustments for baseline values, increased the robustness of our results.

In conclusion, the results of this study, while confirming the benefits of exercise training, show that adding ST to a program of ET and education does not result in further improvement in quality of life for individuals with moderate-to-severe asthma in comparison with ET and education alone. However, the observed improvement in peripheral muscle strength in participants also undergoing ST suggests that it could be a valuable addition to pulmonary rehabilitation programs, particularly for subjects with muscle weakness at the initial assessment.
